# Atypical nested 22q11.2 duplications between LCR22B and LCR22D are associated with neurodevelopmental phenotypes including autism spectrum disorder with incomplete penetrance

**DOI:** 10.1002/mgg3.507

**Published:** 2019-01-04

**Authors:** Karen J. Woodward, Julie Stampalia, Hannah Vanyai, Hashika Rijhumal, Kim Potts, Fiona Taylor, Joanne Peverall, Tanya Grumball, Soruba Sivamoorthy, Hamid Alinejad‐Rokny, John Wray, Andrew Whitehouse, Lakshmi Nagarajan, Jacqueline Scurlock, Sabine Afchani, Matthew Edwards, Ashleigh Murch, John Beilby, Gareth Baynam, Cathy Kiraly‐Borri, Fiona McKenzie, Julian I. T. Heng

**Affiliations:** ^1^ Diagnostic Genomics PathWest Laboratory Medicine Perth Western Australia Australia; ^2^ State Child Development Centre West Perth Western Australia Australia; ^3^ School of Medicine Western Sydney University Penrith South DC New South Wales Australia; ^4^ Genetic Services of Western Australia Perth Western Australia Australia; ^5^ Department of Health Office of Population Health Genomics, Public Health and Clinical Services Division Perth Western Australia Australia; ^6^ Institute for Immunology and Infectious Diseases Murdoch University Perth Western Australia Australia; ^7^ Western Australian Register of Developmental Anomalies Perth Western Australia Australia; ^8^ Spatial Sciences, Science and Engineering Curtin University Perth Western Australia Australia; ^9^ Curtin Health Innovation Research Institute and Sarich Neuroscience Institute Curtin University Crawley Western Australia Australia; ^10^ School of Biomedical Sciences University of Western Australia Perth Western Australia Australia; ^11^ The Harry Perkins Institute of Medical Research QEII Medical Centre Nedlands Western Australia Australia; ^12^ Centre for Medical Research University of Western Australia Nedlands Western Australia Australia; ^13^ Telethon Kids Institute University of Western Australia Perth Western Australia Australia; ^14^ Children's Neuroscience Service Princess Margaret Hospital Subiaco Western Australia Australia; ^15^ School of Paediatrics and Child Health University of Western Australia Perth Western Australia Australia; ^16^ Rural Health West Esperance Western Australia Australia; ^17^ Lockridge Child Development Centre Lockridge Western Australia Australia

**Keywords:** 22q11.2, atypical, autism spectrum disorder, central 22q11.2, duplication, LCR22B to LCR22D

## Abstract

**Background:**

Chromosome 22q11.2 is susceptible to genomic rearrangements and the most frequently reported involve deletions and duplications between low copy repeats LCR22A to LCR22D. Atypical nested deletions and duplications are rarer and can provide a valuable opportunity to investigate the dosage effects of a smaller subset of genes within the 22q11.2 genomic disorder region.

**Methods:**

We describe thirteen individuals from six families, each with atypical nested duplications within the central 22q11.2 region between LCR22B and LCR22D. We then compared the molecular and clinical data for patients from this study and the few reported atypical duplication cases, to the cases with larger typical duplications between LCR22A and LCR22D. Further, we analyzed genes with the nested region to identify candidates highly enriched in human brain tissues.

**Results:**

We observed that atypical nested duplications are heterogeneous in size, often familial, and associated with incomplete penetrance and highly variable clinical expressivity. We found that the nested atypical duplications are a possible risk factor for neurodevelopmental phenotypes, particularly for autism spectrum disorder (ASD), speech and language delay, and behavioral abnormalities. In addition, we analyzed genes within the nested region between LCR22B and LCR22D to identify nine genes (*ZNF74, KLHL22, MED15, PI4KA, SERPIND1, CRKL, AIFM3, SLC7A4, and BCRP2)* with enriched expression in the nervous system, each with unique spatiotemporal patterns in fetal and adult brain tissues. Interestingly, *PI4KA* is prominently expressed in the brain, and this gene is included either partially or completely in all of our subjects.

**Conclusion:**

Our findings confirm variable expressivity and incomplete penetrance for atypical nested 22q11.2 duplications and identify genes such as *PI4KA* to be directly relevant to brain development and disorder. We conclude that further work is needed to elucidate the basis of variable neurodevelopmental phenotypes and to exclude the presence of a second disorder. Our findings contribute to the genotype–phenotype data for atypical nested 22q11.2 duplications, with implications for genetic counseling.

## 1 INTRODUCTION

Chromosome 22q11.2 contains eight highly homologous low copy repeat (LCR) sequences that are named LCR22A to LCR22H and that predispose the region to recurrent deletions and duplications by nonallelic homologous recombination (NAHR) (Edelmann, Pandita, & Morrow, 1999; Shaikh, Kurahashi, & Emanuel, 2001; Shaikh et al., 2000). The four proximal LCRs, LCR22A to LCR22D (A‐D), are implicated in 22q11.2 deletion syndrome (also known as DiGeorge/velo‐cardio‐facial syndrome (DG/VCFS) (OMIM:188400 and OMIM:192430, respectively) and the reciprocal 22q11.2 duplication syndrome (OMIM:608363). The four distal LCRs, LCR22E to LCR22H (E‐H), are smaller in size and are involved in the less frequent distal 22q11.2 deletions and duplications (OMIM:611867) (Mikhail et al., 2014; Wincent et al., 2010).

The typical 3 Mb deletion causing 22q11.2 deletion syndrome occurs between LCR22A and LCR22D and is one of the most frequent pathogenic microdeletions in humans with an incidence of around 1/4,000 live births (Devriendt, Fryns, Mortier, van Thienen, & Keymolen, 1998). Clinical features in patients with typical 22q11.2 deletions are variable and usually include congenital heart defects (mostly conotruncal anomalies), characteristic facial features, immune deficiency, transient neonatal hypocalcaemia, palatal abnormalities (particularly velopharyngeal insufficiency), postnatal growth restriction, hypotonia, developmental delay, intellectual disability, autism spectrum disorder, and behavioral problems (McDonald‐McGinn, Emanuel, & Zackai, 1993). Approximately 8%–10% of individuals with 22q11.2 deletion syndrome have a smaller nested 1.5 Mb deletion, proximally located between LCR22A and LCR22B (Burnside, 2015). The phenotypes of individuals with the 3 Mb typically deleted region (TDR) (A‐D) and the 1.5 Mb deletion (A‐B) are clinically indistinguishable (Carlson et al., 1997), and haploinsufficiency of critical genes, including *TBX1* (OMIM:60254), is the proposed mechanism (McDonald‐McGinn et al., 1993).

Individuals with typical 22q11.2 duplications present with significant inter‐ and intrafamilial variability, with phenotypes ranging from normal or with a mild learning disability to severe congenital malformations (Ensenauer et al., 2003; de La Rochebrochard et al., 2006; Portnoi, 2009; Portnoi et al., 2005). Clinical features can also include more significant degrees of intellectual disability, delayed psychomotor development, growth retardation, and muscular hypotonia (Wentzel, Fernstrom, Ohrner, Anneren, & Thuresson, 2008). Autism spectrum disorder is also a feature of 22q11.2 duplication carriers, with an estimated rate of 14%–25% (Clements et al., 2017; Wenger et al., 2016). Patients diagnosed with the typical 3 Mb duplication, or the nested 1.5 Mb duplication, show no apparent correlation between duplication size and phenotype (Van Campenhout et al., 2012). Furthermore, in contrast to the deletions that are mostly de novo in origin, the majority of duplications are inherited from a mildly affected or clinically normal parent (Clements et al., 2017; Ou et al., 2008; Van Campenhout et al., 2012; Wentzel et al., 2008).

Although 22q11.2 deletions and duplications are reciprocal rearrangements and predicted to occur at the same frequency, the number of duplication cases reported in the literature is considerably less and this is likely due to their milder clinical effect (Portnoi, 2009). A recent case‐cohort study in a Danish population found that although the prevalence of 22q11.2 deletion was within the expected range of approximately 1 in 4,000, the prevalence of 22q11.2 duplications in the population was actually more than twice this number (1 in 1,606) (Olsen et al., 2018). The prevalence of both deletions and duplications was higher among individuals with a neuropsychiatric or developmental disorder than unaffected individuals. However, the overall prevalence of neuropsychiatric disorders was higher in duplication carriers compared with deletion carriers (Olsen et al., 2018).

To date, many individuals have been described with atypical nested 22q11.2 deletions between LCR22B and LCR22D (Clements et al., 2017; Rump et al., 2014; Wentzel et al., 2008). In contrast, very few cases with atypical nested 22q11.2 duplications have been reported (Clements et al., 2017; Diehl, Mu, Batista, & Gunay‐Aygun, 2015; Fan et al., 2007; Fernandez et al., 2009; Pebrel‐Richard et al., 2012; Tucker et al., 2011, 2014). Here, we describe 13 individuals from six families with atypical nested 22q11.2 duplications between LCR22B and LCR22D. We provide molecular and clinical data for each individual and compare this information to the previously reported atypical and typical cases. The number of atypical nested duplication cases is very limited; however, these rare individuals may be able to provide insights into the importance of individual genes within this genomic region and their potential phenotypic contribution. In addition, we undertake gene expression analysis to identify candidate genes within the region LCR22B to LCR22D which are enriched in the brain. Among several candidates, *PI4KA* (OMIM:600286) is a gene highly expressed in fetal and brain tissues, and is disrupted either partially or completely in our subjects. Taken together, our study reinforces varied clinical presentation and neurodevelopmental phenotypes associated with atypical nested 22q11.2 duplications and is consistent with the notion that such duplications represent a risk factor for neurocognitive traits including ASD.

## 2 MATERIALS AND METHODS

### 2.1 Ethical compliance

Informed consent was obtained for this study. The parents of child‐aged patients gave written informed consent to publish clinical information and photographs.

### 2.2 Clinical diagnosis

Individuals in the study were assessed through multiple clinical services and were ascertained to have various combinations of clinical features including developmental delay, intellectual disability, autism spectrum disorder, or multiple congenital anomalies. A standardized procedure for the diagnosis of ASDs and eligibility for Government‐supported therapy provision was applied (Glasson et al., 2008) and described briefly here. For children younger than 12 years of age, the diagnostic assessment is performed by a team of three independent health professionals (pediatrician or psychiatrist, psychologist, and speech/language pathologist). For adolescents (12–17 years) and adults, an assessment must be carried out by a clinical psychologist, a pediatrician (for adolescents) and/or a psychiatrist, with the inclusion of a formalized assessment by a speech‐language pathologist as needed. They were referred for chromosomal microarray testing at the Diagnostic Genomics Department, PathWest, WA. Genomic DNA was isolated from peripheral blood samples according to standard procedures.

### 2.3 Microarray analysis

Microarray was performed using an Infinium^®^ HumanCytoSNP‐12 BeadChip assay according to manufacturer's instructions (Illumina, San Diego, CA). BeadChips were scanned with iScan, with data generated through GenomeStudio (Illumina). Analysis was carried out with KaryoStudio (v4.1), and only copy number variations (CNVs) containing RefSeq genes were interrogated.

### 2.4 Bioinformatic analysis

We mined data from The Functional Annotation of Mammalian Genomes 5 (FANTOM5), a transcriptomic resource generated from RNA analyzed using Cap Analysis of Gene Expression (CAGE) (Consortium et al., 2014). Coordinates for 22q11.2 were imputed into the resource “FANTOM5 PHASE1 FREEZE DPI clusters human (robust)” to identify candidate promoters representing mRNA transcripts within this genomic locus. Data from normalized tag counts from “FANTOM5 Human CAGE Phase1 CTSS‐robust cluster filtered (rle, normalized)” were extracted to construct graphs representing expression profiles for candidate genes (normalized as tags per million (TPM), calculated using the relative log expression (rle). Candidate genes were analyzed for their enriched expression in the nervous system, identified by a statistical evaluation for their ontological classification (UBERON: 0001016). Expression profiles for fetal and adult brain structures derived from human donor samples were also generated for 22q11.2 genes that were enriched in nervous system expression libraries.

### 2.5 GenBank reference sequences

*ZNF74* (NM_001256524.1), *KLHL22* (NM_032775.3), *MED15* (NM_001003891.2), *PI4KA* (NM_058004.3), *SERPIND1* (NM_000185.3), *CRKL* (NM_005207.3), *AIFM3* (NM_144704.2), *SLC7A4* (NM_004173.2), *BCRP2* (NR_037566.1).

## 3 CLINICAL REPORTS

### 3.1 Family 1

#### 3.1.1 Patient 1

Patient 1 is the second child of nonconsanguineous healthy parents with a history of recurrent miscarriage and first child born with bladder exstrophy. Patient 1 was born after an uncomplicated pregnancy and delivered by nonelective cesarean section due to fetal distress. Birthweight was 3,575 g. The parents and child health nurse became concerned from about 20 months of age because of limited speech and interactions. A diagnosis of global developmental delay with autistic features was made at 2 years 6 months of age, and a formal diagnosis of autism spectrum disorder was made at the age of 4 years. At 11 years of age, speech included 7–8 word sentences with limited comprehension and echolalia. Reading skills were approximately at 6‐year age level, he attended the education support unit and there were some behavioral concerns at school. There was no history of seizures. At the time of examination (12 years 6 months), his parents reported him to be very vocal with speech being a relative strength. Height was on the 50th percentile, weight was in the 90th–97th percentile range, and head circumference was on the 98th percentile. A relatively high forehead with widow's peak was present together with posteriorly rotated prominent ears and slightly deep‐set eyes with sparse eyebrows and upslanting palpebral fissures. Motor stereotypies including hand flapping were also present. Microarray analysis identified a paternally inherited 22q11.2 duplication, approximately 730Kb in size, in the region between LCR22B and LCR22D (chr22:20,733,667‐21,462,353 GRCh37/hg19 assembly). Patient 1 also had a de novo duplication of chromosome 19p13.3, approximately 340Kb in size, that was not found in his sister, patient 2, and was of uncertain clinical significance (chr19:1,451,255‐1,788,247 GRCh37/hg19 assembly). Conventional chromosome analysis and fragile *X* testing were also performed, and no abnormalities were detected.

#### 3.1.2 Patient 2

Patient 2 is the third child in family 1 and was delivered at term by elective lower segment caesarean section (LSCS) and weighed 3,060 g. A diagnosis of developmental delay, intellectual impairment, and autism spectrum disorder was made at the age of 3 years. Ongoing issues included obsession with food and pica as well as significant anxiety. There were no reported seizures. At the time of the examination (aged 10 years and 9 months), height was on the 50th percentile, weight was on the 97th percentile, and head circumference was on the 98th percentile. She had a relatively tall forehead, posteriorly rotated ears, and deep‐set eyes. Microarray analysis showed that patient 2 had the same paternally inherited 22q11.2 duplication identified in her brother. Conventional chromosome analysis and fragile *X* testing were also performed, and no abnormalities were detected.

#### 3.1.3 Other family members

The asymptomatic father of patients 1 and 2 had the same duplication as his children (identified as Patient 3 in our study) and a normal male karyotype by conventional chromosome analysis. There was no family history of autistic features or intellectual disability. Patients 1 and 2 have a brother with bladder exstrophy who has not been tested by microarray.

### 3.2 Family 2

#### 3.2.1 Patient 4

Patient 4 was the first child of nonconsanguineous and healthy parents born at term. She was first examined by a pediatrician aged 4 years 6 months due to severe developmental delay. She was nonverbal and had autism spectrum disorder, severe intellectual disability, and epilepsy. Her seizure disorder was comanaged with a neurologist. Her hearing was normal. No cardiac or palatal abnormalities were noted, and she was not dysmorphic. She had short stature and was obese (height 25th percentile and weight >97th percentile). At 12 years and 3 months of age, she attended a special school for children due to her learning and behavioral problems, which included self‐injury. Fragile *X* testing, MECP2 mutation testing, urine and metabolic screens were all negative, and conventional chromosome analysis showed a normal female karyotype. A cerebral MRI was performed aged 6 years 6 months with a nonspecific mild increase in flair signal noted within the periventricular white matter of both parietal lobes. Microarray analysis identified a maternally inherited 22q11.2 duplication, approximately 120Kb in size, in the region between LCR22C and LCR22D (chr22:20,956,906‐21,075,537 GRCh37/hg19 assembly).

#### 3.2.2 Patients 5 and 6

Patients 5 and 6 were presumed monozygotic twin boys. Placental insufficiency and twin‐to‐twin transfusion were noted during the pregnancy. The neonatal ultrasound findings were normal. The twin boys were born at 32‐week gestation by cesarean section.

#### 3.2.3 Patient 5

Patient 5 weighed 1800 g at birth. He was first examined by a pediatrician aged 18.5 months and was diagnosed with global developmental delay The developmental assessment (Huntley, 1996) gave a corrected age of 16.5 months with limited language (age equivalent 9.5 months). He was seen aged 6 years 6 months and diagnosed with mild intellectual disability (IQ score 63, Leiter‐R intellectual assessment test) and autism spectrum disorder. He had only some phrased speech and attended an Educational Support Unit within a mainstream school. He had no reported seizures. He had short stature (3rd percentile) and was overweight for height (50th ‐ 75th percentile). Microarray analysis identified the same maternally inherited 22q11.2 duplication, as his sister and brother.

#### 3.2.4 Patient 6

Patient 6 was a presumed monozygotic twin of patient 5 above. Patient 6 weighed 1,500 g at birth. He was first examined by a pediatrician aged 18.5 months and was diagnosed with global developmental delay (Huntley, 1996). He was seen aged 6 years 6 months, and he was diagnosed with moderate intellectual disability (IQ score 49, Leiter‐R intellectual assessment test) and autism spectrum disorder. Language impairment was moderate and his adaptive behavior was low. He had short stature and obesity (height 25th percentile, weight 97th percentile). Microarray analysis identified the same maternally inherited 22q11.2 duplication, as his sister and brother.

#### 3.2.5 Other family members

The asymptomatic mother of patients 4, 5, and 6 had the same duplication as her three children and was of normal intelligence with short stature and obesity (identified as Patient 7 in our study). There was no family history of schizophrenia or major mental health disorders.

### 3.3 Family 3

#### 3.3.1 Patient 8

Patient 8 was the second of two children born to nonconsanguineous parents of Filipino descent. His mother had well‐controlled gestational diabetes, and he was born at term by nonelective LSCS for failure to progress. His birthweight was 3175 g, his length was 51 cm, and his head circumference was 34 cm. He had speech delay and met DSM IV criteria for a diagnosis of high functioning autism in early childhood. He was examined at age 12 years by a Clinical Geneticist. Clinical findings included mild facial dysmorphism with slightly upslanting palpebral fissures and long luxuriant eyelashes. He was noted to have long narrow hands and fingers and relatively long toes. His growth was in the normal range with height on the 90th percentile, weight between the 50th and 75th percentiles, and head circumference on the 75th percentile. Microarray analysis identified a maternally inherited 22q11.2 duplication, approximately 400Kb in size, in the region between LCR22C and LCR22D (chr22:21,009,596‐21,462,353 GRCh37/hg19 assembly).

#### 3.3.2 Other family members

The asymptomatic mother of patient 8 had the same duplication as her son (identified as Patient 9 in our study). There was no family history of ASD; however, there is a history of mental health issues on the maternal side of the family with patient 8's grandmother (diagnosed with schizophrenia), and uncle (diagnosed with a mild psychosis). His sister has a history of congenital hydrocephalus, childhood stroke, and secondary hemiplegia with mild intellectual disability. The sister was not tested by microarray analysis. Further testing would be required to clarify the basis of the neuropsychiatric differences in this family and a second disorder cannot be excluded.

### 3.4 Family 4

#### 3.4.1 Patient 10

Patient 10 was born vaginally at 41‐week gestation. He was diagnosed with a unilateral cleft lip after birth and has a later diagnosis of a submucous cleft palate. Early motor milestones were achieved at a normal age; however, he had delayed speech and language development thought to be secondary to conductive hearing loss that required speech therapy intervention. He has some fine motor difficulties for which he is seeing an occupational therapist. He has no formal diagnosis of autism, however has some sensory processing issues, a high pain threshold and prefers a structured environment with evidence of mild anxiety if routines are altered. When examined by a Clinical Geneticist at age 5 years 1 month, patient 10 was noted to have a tall forehead, almond‐shaped eyes and upslanting palpebral fissures, long eyelashes, unilateral cleft lip and submucous cleft palate. He had broad fingers of average length. Microarray analysis identified a maternally inherited 22q11.2 duplication, approximately 400Kb in size, in the region between LCR22C and LCR22D (chr22:21,091,640‐21,462,353 GRCh37/hg19 assembly).

#### 3.4.2 Other family members

The mother of patient 10 had the same duplication as her son and was asymptomatic (identified as Patient 11).

### 3.5 Family 5

#### 3.5.1 Patient 12

Patient 12 was investigated aged 2 years 9 months by a neurologist because of involuntary movements and behavioral issues. He was found to have complex motor stereotypies, involuntary movements, and sleep problems with no evidence of seizures. He had macroscopic hematuria, and his kidney ultrasound, renal function, copper levels, and urine metabolic screen were normal. At 2 years 6 months of age, his height was on the 10th–25th percentile, weight was on the 50th percentile, and head circumference was on the 50th–98th percentile. At 4 years and 11 months, he had mood and behavioral issues, and his growth was maintaining its previous trajectory with height on the 25th percentile and weight on the 50th percentile. By age 5 years, he was motor age appropriate with no speech or growth delay and no intellectual disability. Assessment at age 7 years showed development to be within normal limits apart from reading skills. He was found to be more emotional than other boys of his age and demonstrated motor stereotypies, in particular hand flapping. At 7 years and 11 months, his weight and height were on the 50th percentile and his head circumference was on the 75th percentile. He had a normal brain MRI at age 4 years. Microarray analysis identified a 22q11.2 duplication, approximately 400Kb in size, in the region between LCR22C and LCR22D. There is insufficient evidence to determine the pattern of inheritance for their duplication (chr22:21,091,640‐21,462,353 GRCh37/hg19 assembly).

#### 3.5.2 Other family members

There was a family history of anxiety and obsessive–compulsive tendencies. Family members have not been tested for the duplication.

### 3.6 Family 6

#### 3.6.1 Patient 13

Patient 13 is the only child of nonconsanguineous parents of indigenous origin. The pregnancy was complicated by undiagnosed gestational diabetes. He was born at term via spontaneous vaginal delivery weighing 3,400 g. He underwent investigations for hypotonia at the age of 7 months and had a normal head CT at the time. His developmental milestones were delayed, and he was subsequently diagnosed with global developmental delay at the age of three and a half years. His major difficulties were gross motor delay in association with toe walking and speech and language delay. Cognitive abilities at the age of 4 years were found to be in the borderline range (FSIQ 79, WPPSI III). Some early intervention services were accessed in the preschool years. At the age of 14 years, there were ongoing issues with motor stereotypies, in particular hand movements and pacing, that had been present for several years. Furthermore, there were difficulties with social interaction, coping with change as well as anxiety. On physical examination at 14 years, he was tall (height, weight and head circumference on the 98th percentile) with a prominent jaw and forehead. He had reduced dorsiflexion in both ankles. The child was diagnosed with ASD at 16 years of age and is now attending an alternative education program to accommodate his needs. Baseline investigations included fragile X studies, growth hormone studies, IGF1, and thyroid function testing, all of which were unremarkable.

Microarray analysis identified a 22q11.2 duplication, approximately 400Kb in size, in the region between LCR22C and LCR22D (chr22:21,062,271‐21,462,353 GRCh37/hg19 assembly).

#### 3.6.2 Other family members

There is no history of developmental difficulties in the immediate family. The duplication was not maternally inherited; however, the paternal sample was unavailable for evaluation.

## 4 RESULTS

### 4.1 Characterization of atypical nested duplications: no correlation between CNV size and severity of neurodevelopmental traits

**Figure 1:**
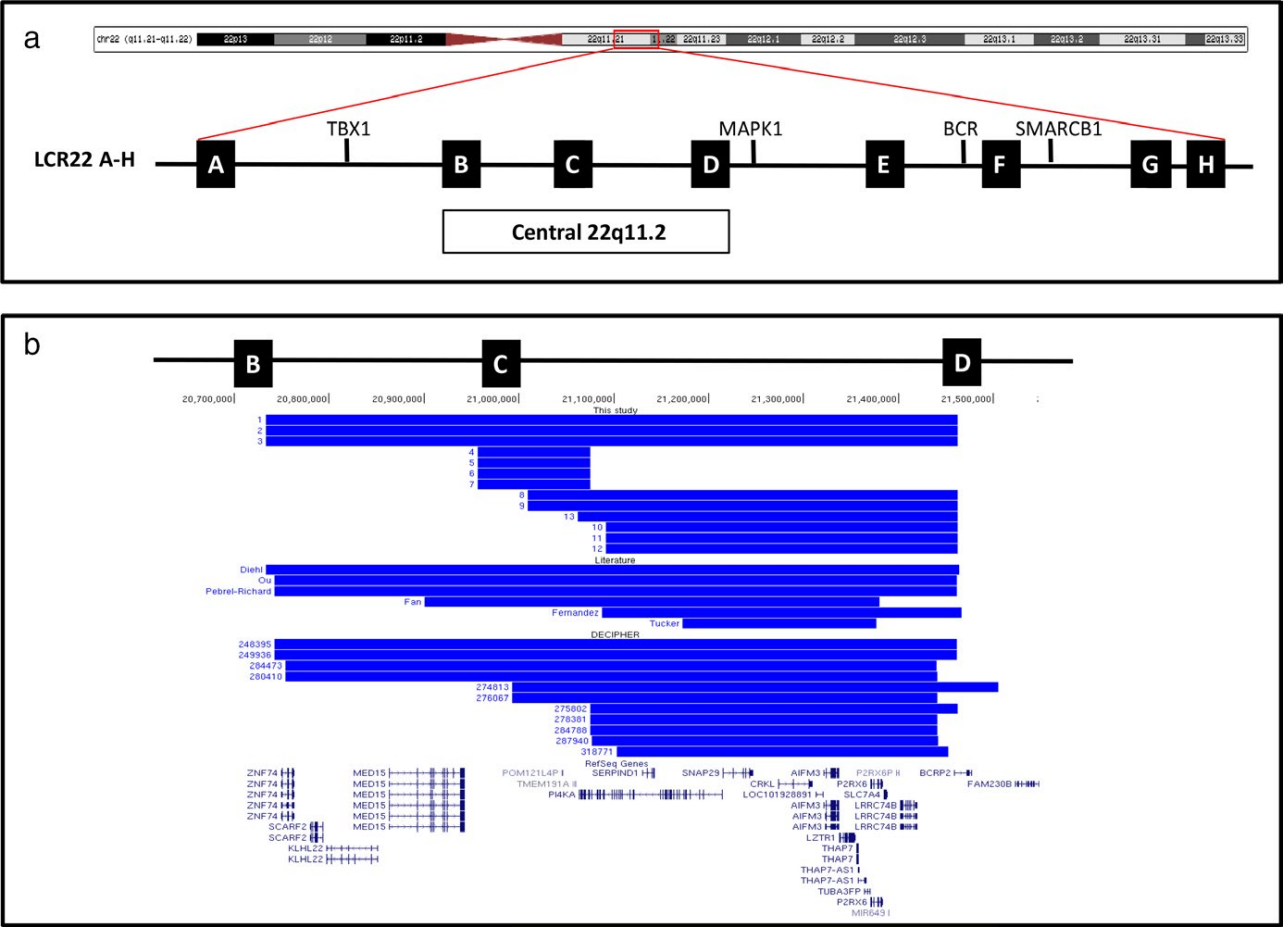
Schematic representation of chromosome 22. (a) The region of interest is shown by a red box on the ideogram of the chromosome. The eight low copy repeat (LCR) sequences located at 22q11.2 are depicted as black boxes (LCR22A‐H). Critical genes are shown and the central 22q11.2 region located between LCR22B and LCR22D. (b) Schematic representation of chromosome 22 duplications identified in patients in this study, reported in the literature and the DECIPHER database (with clinical information and only one CNV). All duplications are located between LCR22B and LCR22D and represented by a horizontal blue line. RefSeq genes in the region are shown. Data based on UCSC Genome Browser 2009 (GRCh37/hg19) Assembly [http://www.genome.ucsc.edu]

Atypical nested 22q11.2 duplications were identified by microarray analysis in 13 individuals from six unrelated families and are shown schematically in Figure 1a–b. The duplications ranged in size from approximately 120Kb to 730Kb. Breakpoint positions also differed between families, with duplications located between LCR22B and LCR22D (B‐D) in family 1, LCR22C and LCR22C/D (C‐C/D) in family 2, and LCR22C/D and LCR22D (C/D‐D) in families 3, 4, 5, and 6. Not all breakpoints coincided with LCR regions. The only other copy number variant identified was a de novo duplication of chromosome 19p13.3 in patient 1 that was not found in his sister, and had uncertain clinical significance. The duplications were found to be inherited in all cases (7/7) where both parental samples were available for testing, with five maternal and two paternal in origin. Confirmed parental carriers were all asymptomatic (families 1, 2, 3, and 4).

**Figure 2:**
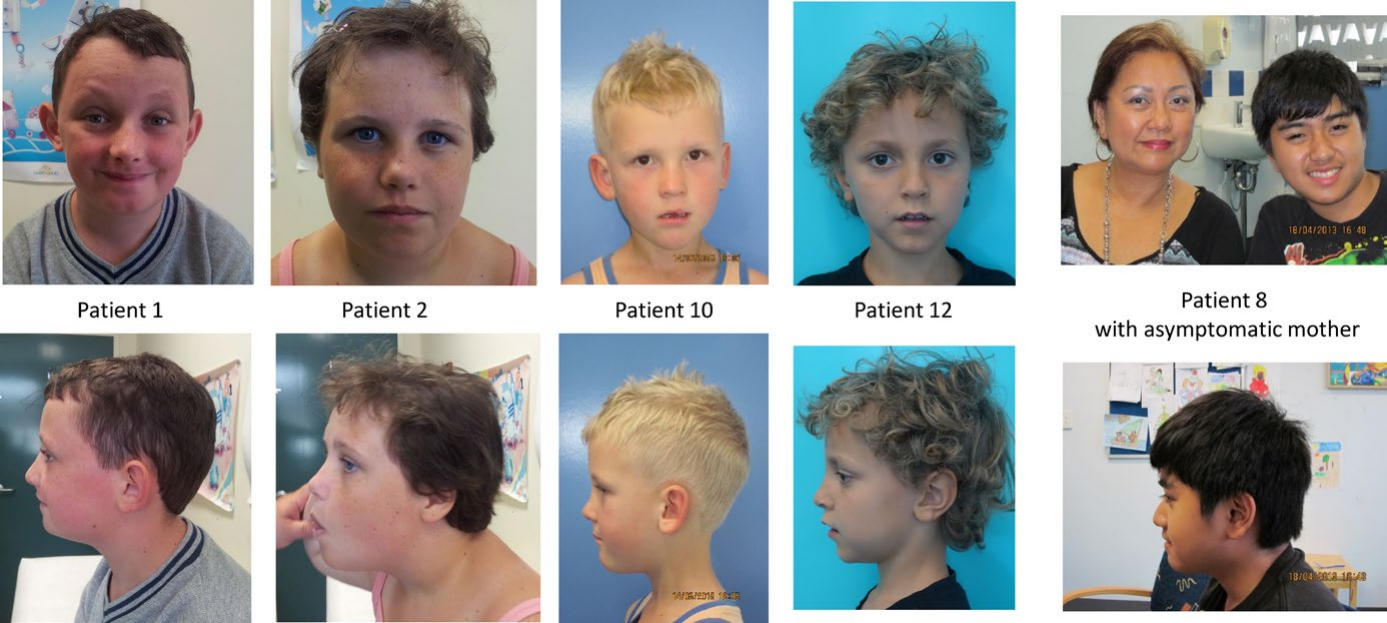
Photographs of patients with central 22q11.2 duplications showing that there is no recognizable facial phenotype. Patient 1 aged 12 years and his sister aged 10 years who have a duplication between LCR22B and LCR22D. Patient 10 aged 5 years who also has a duplication between LCR22C and LCR22D. Patient 12 aged 7 years and 11 months has a duplication in LCR22C and LCR22D. Patient 8 aged 12 years and his asymptomatic mother patient 9 who both have a duplication between LCR22C and LCR22D

Inter‐ and intrafamilial variability in phenotypes was observed among the subjects with atypical nested duplications, ranging from no apparent phenotype in the four parental carriers to severe intellectual disability, developmental delay, and nonverbal autism. Clinical summaries for each patient are shown in Table 1. The majority of the individuals (8/13) had developmental delay/cognitive impairment with most having speech delay and language impairment (7/13). Variability in cognitive impairment was found within the same family, despite having the same size duplication. Many individuals were diagnosed with ASD (7/13 with a formal diagnosis of autism, patients 1, 2, 4, 5, 6, 8, 13) or exhibited features consistent with ASD including stereotypical movements (patient 12) with all symptomatic patients having behavioral problems. Short stature was noted in the three siblings in family 2 (patients 4, 5, 6) and their asymptomatic mother. No significant dysmorphisms were observed, and there was no consistently recognizable facial phenotype. Patients who consented to publication of photographs are shown in Figure 2. A tall forehead was noted in four cases (patients 1, 2, 10, 13) and upslanting palpebral fissures in three (patients 1, 8, and 10). Interestingly, no distinct facial features have been reported for the reciprocal atypical nested deletions, but the common dysmorphic features included upslanting palpebral fissures and a prominent forehead (Burnside, 2015). Hand/foot anomalies were found in two cases (patients 8 and 10). Relative macrocephaly was noted in two siblings in family 1 (patients 1 and 2). Seizures were uncommon (1/13, observed only in patient 4), and no gross abnormalities were reported on brain MRI in all cases examined (data not shown). Palatal defects were also uncommon (1/13, patient 10). Notably, none of our subjects were diagnosed with congenital heart defects.

**Table 1:** Common clinical features identified in individuals with central 22q11.2 duplications

Details	Family 1			Family 2				Family 3		Family 4		Family 5	Family 6
Case No.	1	2	3	4	5	6	7	8	9	10	11	12	13
Sex	M	F	M	F	M	M	F	M	F	M	F	M	M
Age at last evaluation	12 years 6 months	10 years 9 months		12 years 3 months	6 years 6 months	6 years 6 months		16 years		5 years 1 months		7 years 11 months	14 years
Duplication size (Kb)	730	730	730	120	120	120	120	400	400	400	400	400	400
Duplication breakpoints	B to D	B to D	B to D	C to D	C to D	C to D	C to D	C to D	C to D	C to D	C to D	C to D	C to D
Location (hg19)	chr22:20,733,667‐21,462,353			chr22:20,956,906‐21,075,537				chr22:21,009,596‐21,462,353		chr22:21,091,640‐21,462,353		chr22:21,091,640‐21,462,353	chr22:21,062,271‐21,462,353
Other CNVs of possible significance	De novo 19p13.3 duplication	_	_	_	_	_	_	_	_	_	_	_	_
Inheritance	Paternal	Paternal	Unknown	Maternal	Maternal	Maternal	Unknown	Maternal	Unknown	Maternal	_	Unknown	Unknown
Development delay and/or cognitive impairment	Severe global developmental delay, intellectual disability	Developmental delay, intellectual disability	_	Severe intellectual disability, severe developmental delay,	Global developmental delay, moderate intellectual disability	Global developmental delay, mild Intellectual disability	_	Speech delay	_	Early speech and language delay, fine motor difficulties	_	_	Global developmental delay, borderline IQ age 4 years
Psychiatric/Behavior problems	ASD, motor stereotypies	ASD, anxiety	_	Severe ASD, nonverbal at 12 years, self‐injury	ASD, moderate language impairment	ASD, moderate language impairment	_	High functioning ASD	_	Behavioral issues as prefers structure, anxiety with changes in routine (currently no formal diagnosis of ASD)	_	Complex motor stereotypies with repetitive jumping, hand flapping, clenching of hands and face. Mood issues	ASD, variable motor stereotypies: hand movements, pacing, toe walking. Anxious, difficulties with social interaction
Palatal defects	_	_	_	_	_	_	_	_	_	Unilateral cleft lip and submucous cleft palate	_	_	_
Hearing impairment	_	_	_	_	_	_	_	_	_	Conductive hearing loss	_	_	_
Growth delay	_	_	_	Short stature	Short stature	Short stature	Short stature	_	_	_	_	_	_
Macrocephaly	Relative macrocephaly (head circumference 98th percentile)	Relative macrocephaly (head circumference 98th percentile)	_	_	_	_	_	_	_	_	_	_	_
Microcephaly	_	_	_	_	_	_	_	_	_	_	_	_	_
Hypotonia	_	_	_	_	_	_	_	_	_	_	_	_	Hypotonia at 7 months of age
Seizures	_	_	_	Epilepsy	_	_	_	_	_	_	_	_	_
Heart defect	_	_	_	_	_	_	_	_	_	_	_	_	_
Urogenital anomaly	_	_	_	_	_	_	_	_	_	_	_	Macroscopic hematuria. Kidney ultrasound normal	_
Dysmorphism	Tall forehead, posteriorly rotated prominent ears, slightly deep‐set eyes, upslanting palpebral fissures	Tall forehead, posteriorly rotated ears, deep‐set eyes	_	_	_	_	_	Long luxuriant eyelashes, upslanting palpebral fissures	_	Tall forehead, almond‐shaped eyes, long eyelashes, upslanting palpebral fissures	_	_	Tall forehead and prominent jaw
Hand/foot anomalies	_	_	_	_	_	_	_	Long narrow hands and fingers, relatively long toes	_	Broad fingers (average length)	_	_	_
Brain Imaging	_	_	_	Brain MRI with no significant anomalies identified	Neonatal ultrasound with no anomalies detected	Neonatal ultrasound with no anomalies detected	_	_	_	_	_	Brain MRI with no anomalies identified	Head CT scan at 7 months of age with no anomalies detected
Other features	_	_	Asymptomatic	Obese	_	Obese	Asymptomatic, obese	_	Asymptomatic	_	Asymptomatic	_	Reduced dorsiflexion in both ankles

A “‐” sign indicates features which were not present or not detected.

There was no correlation between size of atypical nested duplication and severity of intellectual disability or developmental delay (see Table 1) in symptomatic individuals. The duplication detected in family 2 was the smallest among the patients presented herein (120Kb) and those previously reported. Intrafamilial variability in phenotypes was noted in this family with patient 4 having severe nonverbal ASD, severe intellectual disability, and severe global developmental delay, while her twin brothers (patients 5 and 6) had mild and moderate intellectual disability and language impairment and her mother was clinically unaffected. It is noteworthy that placental insufficiency and twin‐to‐twin transfusion for patients 5 and 6 occurred during pregnancy and represent a significant confound and possible explanation for phenotypic variability with their sister, patient 4. Nevertheless, in all four individuals from family 2, the *PI4KA* gene was the only gene included in the duplicated region and this involved a partial duplication at the 3’ end of the gene. It is possible that this partial duplication may disrupt the normal copy of the gene on this allele. The functional effect of this duplication and the clinical significance is currently unclear. Mutations in *PI4KA* have recently been linked to an autosomal recessively inherited neuronal migration disorder (Pagnamenta et al., 2015). Although the family do not have another CNV, the presence of another genetic modifier or a second disorder cannot be excluded, and so further workup is needed to establish the underlying cause for severe features observed in patient 4.

### 4.2 Genotype–phenotype comparison for symptomatic subjects with atypical nested duplications vs. typical proximal duplications

We investigated the possibility that the clinical features of subjects with atypical nested (LCR22B‐LCR22D) duplications might be distinct from subjects with typical proximal duplications (A‐D). We compared the clinical phenotypes of the nine symptomatic individuals in this study with other known atypical nested duplications (six previously reported and 11 cases in the DECIPHER database with clinical details and only one CNV present (https://decipher.sanger.ac.uk) (see Supporting information Table S1). These atypical nested 22q11.2 duplications (*n* = 26) were then compared to a study of typical 22q11.2 duplications (*n* = 37) (see Table 1) (Wenger et al., 2016) and led to the following findings. Firstly, we observed that the majority of both duplications were inherited, with only one nested duplication (case #318771 of DECIPHER) reported to be de novo. Secondly, we found a relatively high frequency of developmental delay/cognitive impairment in the atypical nested 22q11.2 duplication cases (81%), a finding which is consistent with a previous study of the larger typical duplication cases (Wentzel et al., 2008). Interestingly, the incidence of ASD and behavioral problems associated with nested atypical duplication in all duplication carriers (asymptomatic parents and their symptomatic children) in our study is 46% (6/13), which is consistent with autism as a feature of 22q11.2 typical duplication carriers. However, our small sample size and reporting bias represent significant confounds in our estimation. Thus, we calculated the incidence of ASD in symptomatic individuals in our study together with those reported in the literature and from the DECIPHER database to observe a prevalence rate of 31% (8/26; see Table 2), including two DECIPHER patients (#249936 and #276067). Our finding is in agreement with estimated rates of 14%–25% for ASD diagnosis in symptomatic individuals with a typical duplication (Wenger et al., 2016).

**Table 2:** Comparison of clinical features in atypical central 22q11.2 duplications and typical proximal duplications

Type of duplication	Central 22q11.2 duplications (B‐D)				Proximal 22q11.2 duplications (A‐D)
Cohort of symptomatic patients	This study (*n* = 9, 6 families)	Literature (*n* = 6)	Decipher (*n* = 11)	TOTAL (*n* = 26)	Wenger et al. (2016) (*n* = 37)
Inherited	7/7 tested	4/4 tested	8/9	19/20 inherited (95%)	
De novo	0	0	1	1/20 (5%)	
Development delay and/or cognitive impairment	8	3	10	21/26 (81%)	
Psychiatric/Behavior problems	9	0	3	12/26 (46%)	
ASD	7	0	2	9/26 (35%)	14%–25%
Palatal defects	1	2	0	3/26 (12%)	
Hearing impairment	1	0	0	1/26 (4%)	6/37 (16%)
Growth delay	3	1	2	6/26 (23%)	
Hypotonia	1	1	0	2/26 (8%)	10/37 (27%)
Seizures	1	0	3	4/26 (15%)	7/37 (19%)
Heart defect	0	0	2	2/26 (8%)	9/37 (24%)
Urogenital anomaly	0	1	1	2/26 (8%)	

Only symptomatic individuals are included in this table. Central duplications cases included those between LCR22B and LCR22D described in this study, published in the literature (Diehl et al., 2015; Fan et al., 2007; Fernandez et al., 2009; Ou et al., 2008; Pebrel‐Richard et al., 2012; Tucker et al., 2014) and within the DECIPHER database with clinical information and one CNV (https://decipher.sanger.ac.uk). Typical proximal duplication cases included those 3 Mb in size between LCR22A and LCR22D (Wenger et al., 2016).

The incidence of seizures was not appreciably different between atypical (15%) and typical duplications (19%) (Wenger et al., 2016). In contrast, we observed reductions in the frequency of association with hypotonia (8% atypical vs. 27% typical) and hearing impairment (4% vs. 16%) (Wenger et al., 2016). We found that nested duplications in this study were not associated with congenital heart disease, although there are two such cases reported in DECIPHER (#280410 and #278381). Despite this, nested duplications remain less frequently associated with congenital heart defects when compared to typical A‐D duplications (8% atypical nested vs. 24% typical) (Wenger et al., 2016); however, we acknowledge that our results are based on a small sample size. Nevertheless, this observation suggests that nested atypical duplications could present with reduced penetrance for significant clinical phenotypes such as cardiac defects, hypotonia, and hearing impairment when compared to the larger typical duplications. It is interesting to note that this is not the case for the incidence of neurodevelopmental traits, which are relatively prevalent for both typical and atypical nested cases. This is supported by a recent report describing similar rates of ASD in individuals with typical duplications and atypical nested duplications B‐D (Clements et al., 2017). Therefore, it is plausible that the critical genes associated with these neurodevelopmental phenotypes including ASD may be located within the nested region between LCR22B and LCR22D.

### 4.3 Identification of genes enriched in the nervous system that are located within the nested LCR22B to LCR22D region

**Figure 3:**
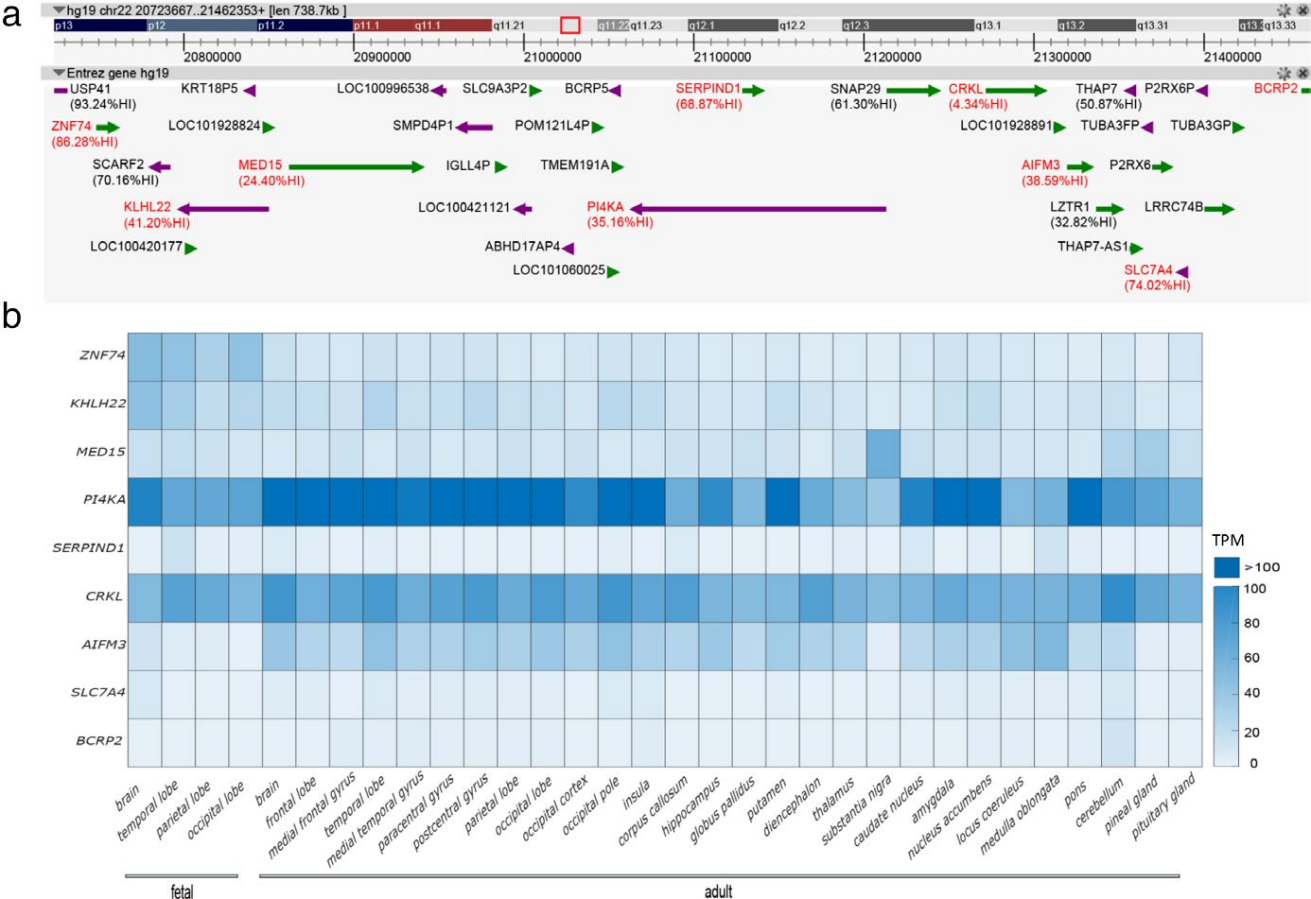
Identification of neuronal genes within 22q11.2 LCR22B to LCR22D. (a) Analysis of 22q11.2 genes (hg19:chr22:20733667‐21462353) with FANTOM5. Of the 88 CAGE defined promoters within 22q11.2, 23 promoters showed significant enrichment for expression in “nervous system”‐related samples (sample ontology enrichment analysis). Genes enriched in the nervous system (highlighted in red) are identified by the UBERON anatomical term “nervous system.” Where available, Haploinsufficiency Index (%HI) scores are provided as an indication of the potential deleterious effect of candidate gene loss. (b) Heat map representing expression levels for *ZNF74* (NM_001256524.1), *KLHL22* (NM_032775.3), *MED15* (NM_001003891.2), *PI4KA* (NM_058004.3), *SERPIND1* (NM_000185.3), *CRKL* (NM_005207.3), *AIFM3* (NM_144704.2), *SLC7A4* (NM_004173.2), *BCRP2* (NR_037566.1). Data from normalized tag counts from “FANTOM5 Human CAGE Phase1 CTSS‐robust cluster filtered (rle, normalized)” were extracted to construct graphs representing expression profiles for candidate genes (normalized as tags per million (TPM), calculated using the relative log expression (rle). Gene expression profiles data are provided in Supporting information Table S2

We hypothesized that genes within the nested duplication region may be relevant to brain development and ASD traits. Fourteen RefSeq genes lay within LCR22B‐LCR22D, with four genes located in the LCR22B‐LCR22C region including *ZNF74* (zinc finger protein 74) (OMIM:194548), *SCARF2* (scavenger receptor class F member 2) (OMIM:613619), *KLHL22* (kelch‐like family member 22) (OMIM:616262), and *MED15* (mediator complex subunit 15) (OMIM:607372), while 10 genes encompassed in the LCR22C‐LCR22D region including *PI4KA* (phosphatidylinositol 4‐kinase, catalytic, alpha), *SERPIND1* (serpin peptidase inhibitor, clade D (heparin cofactor), member 1), *SNAP29* (synaptosomal‐associated protein, 29 kDa) (OMIM:142360), *CRKL* (v‐crk avian sarcoma virus CT10 oncogene homolog‐like) (OMIM:602007), *AIFM3* (apoptosis inducing factor, mitochondria associated 3) (OMIM:617298), *LZTR1* (leucine‐zipper‐like transcription regulator 1) (OMIM:600574), *THAP7* (THAP domain containing 7) (OMIM:609518), *P2RX6* (purinergic receptor P2RX6) (OMIM:608077), *SLC7A4* (solute carrier family 7 member 4) (OMIM:603752), and *LRRC74* (leucine‐rich repeat containing 74B) (Figure 3a).

We sought evidence for the expression of 22q11.2 genes in tissues of the nervous system, particularly those within the LCR22B‐LCR22D interval. We surveyed the gene expression database FANTOM5 to identify central 22q11.2 genes which were significantly enriched in expression within the nervous system, identified with the UBERON (Dahdul et al., 2012) classification term UBERON:0001016. Through this approach, we identified nine genes (*ZNF74*,* KLHL22*,* MED15*,* PI4KA*,* SERPIND1*,* CRKL*,* AIFM3*,* SLC7A4*, and *BCRP2*) which were enriched in the nervous system. Furthermore, each of these nine genes displayed distinct patterns of expression (Figure 3b). For example, mRNA transcripts for *ZNF74* and *KHLH22* were more abundant in fetal vs. adult tissue. Conversely, *PI4KA* and *AIFM3* were more prominently expressed in adult brain tissues. Genes such as *CRKL* and *MED15* displayed relatively constant levels of expression across fetal and brain tissues. In contrast, *SERPIND1*,* SLC7A4*, and *BCRP2* are very weakly expressed in brain tissues. Notably, *PI4KA* is among the most highly expressed genes within this interval and is prominently detected in fetal and adult brain, with particularly high levels in adult cerebral cortical tissues. In contrast, *SERPIND1* is less attractive as a candidate gene as it is very weakly expressed in brain tissues.

## 5 DISCUSSION

Our study reinforces the notion that atypical nested 22q11.2 duplications are associated with a broad phenotypic spectrum, ranging from clinically normal to severe developmental delay with profound intellectual disability. We confirm incomplete penetrance and no association between duplication size and clinical severity; the family with the smallest duplication described to date includes patients with the most severe clinical presentation. We acknowledge that there is ascertainment bias in the identification of atypical duplications in this group of patients. However, it is likely that cases of nested 22q11.2 duplications with significant and profound neurocognitive disorders are a consequence of additional causative factor(s) that augment the severity of the clinical phenotypes, and thus in these settings, great caution should be applied when considering nested duplications as the sole or even predominant contributing factor. What is clear is that while typical 22q11.2 duplication carriers show elevated (14%–25%) rates of ASD (Wenger et al., 2016), a significant number of individuals show no signs of ASD or related developmental concerns. Thus, while we cannot rule out the possibility that nested atypical duplications represent a benign variant, our study supports the notion that atypical nested 22q11.2 LCR22B‐LCR22D duplications are associated with an increased risk for neurodevelopmental phenotypes particularly ASD. Furthermore, our conclusion is consistent with a recent study reporting that the incidence of ASD is similar in both typical and the nested atypical duplication cases, further suggesting that the nested duplication and its genes constitute the minimal interval which is relevant to neurodevelopmental phenotypes including ASD (Clements et al., 2017).

In highlighting our patients harboring 22q11.2 duplications (LCR22B‐LCR22D) nested within the typical 22q11.2 region (LCR22A‐LCR22D), our aims were to expand the reported 22q11.2 phenotypic spectrum as well as investigate candidate genes that may be associated with distinct clinical features. We found that common symptoms associated with atypical central 22q11.2 duplications include developmental delay/cognitive impairment, autism spectrum disorder, and behavioral problems, thereby suggesting that candidate gene(s) for such neurological traits may be located in the nested LCR22B‐LCR22D interval. When the gene content of the central region was analyzed using FANTOM5, several candidates for neurodevelopmental phenotypes, including *PI4KA* and *AIFM3*, were found to be enriched in the nervous system. *PI4KA* has been associated with schizophrenia susceptibility (Vorstman et al., 2009). Also, compound heterozygous mutations in the *PI4KA* gene were identified in tissue samples from three fetuses with multiple congenital abnormalities, conceived by unrelated parents of European descent, with perisylvian polymicrogyria (PMG), cerebellar hypoplasia, and arthrogryposis (Pagnamenta et al., 2015). Interestingly, *PI4KA* was included in the duplicated regions of all the families presented, either completely or partially, and it was the only gene located within the duplicated region in one family with three severely affected individuals and an asymptomatic carrier mother. Thus, our analysis implicates a role for the *PI4KA* gene to underlie ASD traits, and prioritizes this gene for further study in brain development.

In contrast to the prevalence of developmental delay/cognitive impairment, autism spectrum disorder, and behavioral problems in our subjects, we found no incidence of cardiac defects in any of our symptomatic patients with central duplications. We therefore postulate that the responsible genes for cardiac defects are likely to lie beyond the LCR22B to LCR22D interval. Of note, *TBX1* is a well‐characterized gene within the LCR22A‐LCR22D interval that has a critical role in the abnormal phenotype associated with 22q11.2 genomic disorders (Liao et al., 2004; Vitelli, Morishima, Taddei, Lindsay, & Baldini, 2002; Yagi et al., 2003), especially the cardiac defects, and lies upstream of the LCR22B to LCR22D interval. Given that significantly fewer of our subjects presented with cleft palate, hypotonia, and hearing impairment, it is plausible that the responsible genes for such factors may also lie beyond the central LCR22B to LCR22D interval.

The variable expressivity and incomplete penetrance observed in this study are a common finding with CNVs associated with neurodevelopmental disorders, and the underlying mechanisms are not well understood (Vorstman et al., 2009). Genetic background, modifier genes, epigenetic changes, and environmental factors may have an important role. Another factor that has been reported to influence the penetrance of CNVs, in particular for the expression of intellectual impairment in 22q11.2 deletion patients, is the level of parental intelligence (Klaassen et al., 2016). Given that environmental factors are important during early childhood but become less significant over time, inherited genetic factors associated with higher levels of education were proposed to modulate cognitive outcome and explain the variable penetrance (Klaassen et al., 2016). While such issues were not formally assessed in our study, the impact of environmental factors as well as additional genetic mutations contributing to neurodevelopmental traits in 22q11.2 duplication carriers remains to be clarified.

Variability in expressivity of recurrent CNVs can also be explained by a two‐hit hypothesis and the presence of a second modifying aberration (Aggarwal & Morrow, 2008; Girirajan et al., 2010, 2012; Li, Tekin, Buch, & Fan, 2012). It has been suggested that CNVs outside the 22q11.2 region may include genes that modify risk for heart defects in some patients with 22q11 deletion syndrome (Mlynarski et al., 2016). Although only patient 1 in this study had an additional CNV identified, we cannot exclude the co‐existence of other mutations or CNVs that may directly influence the interpretation of results. Patient 1 also had a chromosome 19p13.3 duplication. This region of 19p13.3 contains a number of genes, and a deletion of this region has been implicated in a neurodevelopmental phenotype (Palumbo et al., 2016). Currently, there is no evidence to suggest that the 19p13.3 duplication is clinically significant and that the involved genes are triplosensitive; furthermore, this patient did not have a significantly more severe phenotype than his younger sister (patient 2), who did not have the 19p13.3 duplication. In another case, a patient is described with a central 22q11.2 duplication already recognized to have more than one genetic aberration (Diehl et al., 2015). This 3‐year‐old female patient had an additional missense mutation in the *SALL4* gene and presented with normal cognition and multiple skeletal anomalies, including left radioulnar synostosis, thumb aplasia, rib abnormalities, hypoplasia of the humeral and femoral epiphyses, and butterfly vertebrae. Interestingly, these skeletal abnormalities had not previously been associated with *SALL4* mutations or 22q11.2 duplications. Additional pathogenic mutations contributing to the severity of the neurodevelopmental phenotypes in 22q11.2 duplication carriers have to be considered. Demily and colleagues described two patients with distal 22q11.2 duplications and severe clinical presentations explained by additional pathogenic mutations (Demily et al., 2018). We cannot exclude this possibility and further work is required, particularly for those individuals with the more severe phenotypes.

## 6 CONCLUSION

This study extends the phenotypic spectrum of nested atypical 22q11.2 duplications between LCR22B and LCR22D with variable expressivity and an association with neurodevelopmental phenotypes including autism spectrum disorder and behavioral problems. In addition, we have analyzed the gene content of the region LCR22B‐LCR22D to identify multiple genes which are highly expressed in the nervous system, and prioritize *PI4KA* as a candidate gene important to neuronal development. Furthermore, we find that atypical nested 22q11.2 duplications show incomplete penetrance, consistent with the notion that additional pathological mechanisms or genetic factors likely modulate or may even explain their clinical presentation in some cases.

## CONFLICT OF INTERESTS

None declared.
